# Experimental Tuberculosis, Human and Bovine, in the Domestic Animals1Presented at the Thirty-seventh Annual Meeting of the American Veterinary Medical Association, held at Detroit, Mich., September 4, 1900.

**Published:** 1901-01

**Authors:** R. R. Dinwiddie

**Affiliations:** Arkansas Agricultural Experiment Station


### EXPERIMENTAL TUBERCULOSIS, HUMAN AND BOVINE, IN THE DOMESTIC ANIMALS.[1]

By R. R. Dinwiddie, M.D., V.S.,
ARKANSAS AGRICULTURAL EXPERIMENT STATION.

(Concluded from page 722.)

*Inoculation with Bovine Cultures.* The cultures employed in this test had the following designations and history:

Culture, Bovine I. Twelfth transfer, twenty-seven days old, twenty-three months from guinea-pig.

Bovine I. (Smith).[2] Second transfer in my laboratory, twenty-seven days old, five years from animal.

[1] Presented at the Thirty-seventh Annual Meeting of the American Veterinary Medical Association, held at Detroit, Mich., September 4, 1900.

[2] Cultures supplied by Professor Smith and described in the Journal of Experimental Medicine, vol. iii., Nos. 3 and 4, p. 454. Glycerin-agar series.

Bovine III. (Smith). Third transfer in my laboratory, twenty-seven days old, three years from animal.

Bovine VIII. (Smith).[1] Fourth transfer in my laboratory, (tenth transfer in all), eighteen days old, nine months from animal.

All cultures at the time of use were growing satisfactorily on glycerin serum. It will be noticed that three of these cultures had been grown on artificial media much longer than the sputum series. However, the results showed that their virulence could not have been much lessened on this account.

Four healthy six-months' old pigs were inoculated in the same manner as in the sputum series on April 6, 1900. They died on the forty-first, forty-eighth, fifty-second, and sixty-eighth days after inoculation, generally without marked ante-mortem symptoms, although all rapidly emaciated in the last two or three weeks. The pig receiving the most recently isolated culture, Bovine VIII., was the first to die.

In all the tubercular changes in the peritoneal cavity were similar to those in the sputum series, but much more extensive, and the lymph nodes were generally involved. In two there was, in addition, a thickening and reddening of the omentum and considerable reddish-colored fluid effusion. In three the lungs were densely crowded with translucent or yellow tubercles, while in a fourth these were not evident, although a scraping from the cut surface of the lung showed numerous tubercle bacilli. This case was also peculiar from the presence of a large pleuritic effusion and marked atrophic degeneration of liver and spleen. In all these pigs tubercle bacilli were found in large numbers in the lung and lymph glandular lesions. This test, which was uncomplicated with extraneous disease, unmistakably proves the greater virulence of the bacilli of bovine origin for pigs.

Experiment III. *Comparative Tests on Sheep. Sputum Cultures.* Four sheep of from three to five months old were inoculated in July, 1899. The mode of inoculation and cultures used were the same as in the last experiment. The animals were kept constantly penned. One died at the end of a month, after two weeks of evident illness. Dissection revealed an extensive pneumonia involving the right lung, abscesses containing thick, greenish pus near the seat of inoculation, and an extensive distribution of minute seed-like tubercles over the abdominal serosa. There was no positive identification of tubercular changes in the thoracic cavity, and

1 Previous growth in Smith's laboratory on dog's serum.

death apparently resulted from the incidental pneumonic complication. The place of this animal was supplied by another, inoculated with the same culture.

All these sheep were in fair condition when killed about one year later, namely, in June and July, 1900. They reacted distinctly to the tuberculin test, applied a week or two before being killed.

The necropsic lesions were in general confined to the abdominal cavity—densely calcareous tubercles, reaching to the size of a small pea, in varying numbers throughout the serosa; in two cases some local peritonitis, with abscesses containing thick, greenish pus near the site of inoculation. One only showed extension of the disease to the thoracic cavity. In this case the peribronchial lymph nodes were much enlarged, caseous, and calcareous, presenting an appearance exactly similar to what is commonly found in the spontaneously acquired disease of cattle. Tubercle bacilli were in general difficult of demonstration in these old, calcareous nodules. Nevertheless, in one such nodule they were found in large numbers.

I should note also the fact that in two of these sheep minute grayish points were scattered in small numbers throughout the lung substance. These I have not been able to identify as tubercular structures.

*Tests of Bovine Cultures on Sheep.* To compare with the above experiment four sheep have been inoculated with bovine cultures, three of which have been freshly isolated from diseased cattle; but these latter tests I must omit from this communication, as they are still uncompleted.[1] The other has been described in my previous report, but I may repeat here the main facts. Death resulted in four months after inoculation with a five-weeks' old culture (six months isolated from the guinea-pig), with enormous development of tubercular nodules in the peritoneum and in the lungs.

Judging from this one test the excess of virulence of the bovine variety of bacillus apparently holds good also for sheep.

EXPERIMENT IV. *Tests of Sputum Cultures on Cattle.* The feeble susceptibility of cattle toward sputum cultures has been already well shown by the experiments of Smith and of Frothingham as well as by experiments of my own previously reported. The effects of inoculation of cultures of human origin were in my first experiments so insignificant that I decided to make some further tests of the degree of this insusceptibility—to see whether or not

[1] Two of these sheep died within a month of inoculation with generalized tuberculosis. The other died four days after inoculation without anatomical lesions.

it was possible to induce a fatal or at least progressive disease in cattle by infection with the bacilli of human phthisis.

In this experiment there were employed five cattle, ranging in ages from seven to fifteen months. The cultures, Sputum I., II., III., and IV., have been described in the experiment on sheep. Large doses, about two pin-head-sized pieces made into a suspension in twelve cubic centimetres of sterile normal salt solution, were injected into the peritoneum. One calf also received an injection into the lung. The inoculations were made in July and September, 1899, and the cattle kept most of the time on pasture, provided with shelter, and liberally fed. The same cattle had been used in previous tuberculosis inoculations with sputum, but the results of the present tests are not, in my opinion, vitiated on this account. However, in order to make the record complete, I have arranged in a condensed form the previous treatment to which these animals were subjected (more fully given in *Bulletin* 57, *Arkansas Agricultural Experiment Station*) as well as the details of the present experiment:

Calf K (ten months old).

[*December* 7, 1898. Culture, Sputum I. (dead culture) in right lung.

*April* 20, 1899. Tuberculin test negative.]

*May* 3, 1899. Culture, Sputum I. in left lung.

*July* 12, 1899. Tuberculin test negative.

July 28, 1899. Culture, Sputum I. in peritoneum.

*September* 22, 1899; *December* 29, 1899; *April* 16, 1900. Tuberculin tests negative.

*August* 7, 1900. In good condition; inoculated with bovine tubercle bacilli culture. (Culture, Bovine VI. in peritoneum.)[1]

Calf P (three months old).

[*December* 2, 1898. Inoculated with tubercular nodules from Calf M (which had been inoculated with sputum cultures).

*February* 24, 1899; *April* 20, 1899; *July* 12, 1899. Tuberculin tests negative.]

*July* 28, 1899. Culture, Sputum II. in peritoneum.

*September* 22, 1899; *December* 29, 1899; *April* 6, 1900. Tuberculin tests negative.

*August* 3, 1900. In good condition; used for inoculation with bovine tubercle bacilli. (Culture, Bovine V. in peritoneum.)[2]

1 When killed, November 27, 1900, this animal showed extensive abdominal lesions referable to the last inoculation. Older calcareous lesions were not discoverable.

2 This calf died September 24, 1900, with enormous recent tubercular lesions in abdomen and thorax. No older calcareous lesions discoverable.

Calf T (two months old).

[*February* 15, 1899. Inoculated with Sputum IV. in peritoneum.

*February* 22, 1899. Tuberculin test negative.

*April* 20, 1899. Tuberculin test dubious.

*July* 12, 1899. Tuberculin test negative.]

*July* 27, 1899. Culture, Sputum III. in peritoneum.

*September* 22, 1899. Tuberculin test dubious.

*December* 29, 1899; *April* 6, 1900. Tuberculin tests negative.

*August* 13, 1900. In fair condition; killed for examination. No tubercular lesions found.

Calf Q (three months old).

[*December*, 1898; *January*, 1899. Fed, on several occasions, cultures of tubercle bacilli from sputum.

*February* 22, 1899; *April* 18, 1899. Tuberculin tests negative.]

*May* 3, 1899. Cultures, Sputum II. and III. in peritoneum.

*July* 12, 1899. Tuberculin test positive.

*September* 21, 1899; *December* 29, 1899. Tuberculin tests negative.

*April* 6, 1900. Tuberculin test negative. In good condition and apparently free from disease. Used for swine-pest antitoxin work.[1]

Calf U (three months old).

[*April* 7, 1899. Human tubercular(?)[2] glands of neck in peritoneum.

*April* 20, 1899; *July* 12, 1899. Tuberculin tests negative.]

*September* 8, 1899. Culture, Sputum IV. in peritoneum.

*September* 22, 1899. Tuberculin test positive.

*December* 29, 1899. Tuberculin test dubious.

*April* 6, 1900. Tuberculin test negative. Calf now in good condition, without indications of disease. Used for swine-pest antitoxin experiments.[3]

The successive events in the case of each animal are here chronologically arranged. It will be seen that the earlier inoculations were almost certainly without permanent effect, since tuberculin injections failed to elicit a reaction in four animals and gave only an uncertain reaction in the other, which was followed later by

[1] At this date (December, 1900) is still apparently sound.

[2] Diagnosticated clinically as tubercular, but microscopical finding and result of guinea-pig inoculation negative.

[3] When killed for examination on December 3, 1900, this animal was found to be free from all tubercular lesions.

failure to react. In two animals the possibility of an immunizing effect from these first inoculations must be admitted, since living tubercle bacilli, although in small doses, were injected. In the others this factor may be excluded even as a possibility.

The result, therefore, of this last experiment is to show the innocuousness of tubercle bacilli of human sputum toward cattle. It is almost certain—as certain, indeed, as the tuberculin test—that none of these cattle, now a year after inoculation, has any progressive tubercular lesions within them or even such as we find in the mildest form of the natural disease. In arriving at this conclusion I have been content, in general, to abide by the result of the tuberculin test, carefully applied, and the apparently thrifty condition of the animals. One only, the thinnest of the lot, was killed for post-mortem examination, and this one showed no traces of disease, either local or general. A positive tuberculin reaction was obtained in only two cases, in another a doubtful reaction, both two months after inoculation; later tests were negative in results. We may infer from this, which is in agreement with previous similar tests, that inoculation of sputum cultures in cattle is followed by a local reaction due to a limited growth of the injected bacilli, or, in some cases, merely to their presence without further vegetation. An involvement of the adjacent lymph nodes may occur in some cases. In others not even this limited extension from the seat of inoculation can be demonstrated. The minute calcareous tubercles of the omentum, however, may contain living tubercle bacilli, either those originally injected or their progeny, many months after inoculation and after the animal has ceased to react to tuberculin injections.

The results of such inoculations are undoubtedly greatly influenced by the conditions under which the animals are kept, hygienic or otherwise, but it is not possible to place any accurate estimate on this factor. Under the conditions in which my experimental stock were kept I am satisfied that no permanent or contagious tubercular disease can be induced in cattle by the bacilli of human pulmonary phthisis.

Comparative tests with bovine cultures were not included in this experiment. Several such tests have previously been made by me and reported, the results in general being in accord with those obtained in the well-known experiments of Smith. I have, however, inoculated two of the cattle of Experiment IV. with recently isolated bovine cultures and now await the result of this test, interesting from several points of view.

My experiments on cats are also of recent date and the results still undetermined. Two only have been inoculated, one with each variety of the bacilli.

Guinea-pigs have been used only as witnesses to the larger animals, to test, as a matter of routine merely, the vitality of the cultures employed.

In reviewing the results of the foregoing experiments, considered in their relation to agricultural or veterinary science rather than to comparative pathology, I may formulate the following conclusions, which seem to be thoroughly established by the evidence which we now possess:

1. Toward tubercle bacilli of human consumption pigs, sheep, and cattle show a susceptibility in the order named. Pigs may obtain a genuine progressive tuberculosis by infection from man, while cattle are practically insusceptible.

2. As regards tubercle bacilli of bovine tuberculosis all three species are alike susceptible to the inoculation disease.

3. The excess of virulence of the bacilli of cattle tuberculosis over that of human consumption, previously demonstrated in cattle and rabbits, holds good also for pigs and sheep—for all susceptible animals, practically, on which the test has so far been made.

I may add to this, in conclusion, an opinion of my own, forced upon me largely by the experience I have had in these studies of experimental tuberculosis in animals, namely, that the conditions of environment, in their influence on the initiation and progress of tuberculosis in cattle and in mankind as well, have been and are even now greatly underestimated. Among the unfavorable influences I attribute much the greatest importance to the breathing of vitiated air—disregard of ventilation—in houses and stables.

---

THE USE OF TUBERCULIN FOR THE EARLY DIAGNOSIS OF TUBERCULOSIS IN MAN. Prof. B. Fränkel, in a recent article (*Berliner klinische Wochenschrift*, 1900, No. 12, p. 255), calls attention to the value of tuberculin in the early diagnosis of tuberculosis in man. He discusses the method by which tuberculin operates and the technique of its application. For adults the dose recommended is 1 mg.; for children, ½ mg. of the old (original) tuberculin, such as is used for testing cattle. The temperature should be taken every three hours after injection. An elevation of 0.5° C. indicates a reaction. Fränkel does not fear any evil results from the test, which may be applied to patients in the hospital, at home, or in the dispensary service. False results are relatively rare. It is advised that the tuberculin test should be used in all cases in patients admitted to hospitals for the treatment of diseases of the lungs if a diagnosis cannot be made by finding tubercle bacilli in the sputum. In these cases the test is of great value, and also in those diseases that may occur in the early stages of tuberculosis, as chlorosis, in scrofula in children, and in surgical tuberculosis.

